# Concurrent Coxsackievirus A6 Infection and Kawasaki Disease: A Case Report

**DOI:** 10.3390/reports7040098

**Published:** 2024-11-15

**Authors:** Jiratchaya Puenpa, Noree Saelim, Nasamon Wanlapakorn, Sumeth Korkong, Ritthideach Yorsaeng, Yong Poovorawan

**Affiliations:** 1 Center of Excellence in Clinical Virology, Department of Pediatrics, Faculty of Medicine, Chulalongkorn University, Bangkok 10330, Thailand; jiratchaya.pu@gmail.com (J.P.); nasamon.w@chula.ac.th (N.W.); sumeth.kor@gmail.com (S.K.); ritthideach.yor@gmail.com (R.Y.); 2 Department of Pediatrics, Phitsanulok Bangkok Hospital, Phitsanulok 65000, Thailand; sunnoway@yahoo.com; 3 The Royal Society of Thailand, Sanam Sueapa, Dusit, Bangkok 10300, Thailand

**Keywords:** Kawasaki disease, coxsackievirus A6, herpangina, hand foot mouth disease

## Abstract

**Background and Clinical Significance:** Kawasaki disease (KD) is an acute febrile vasculitis that primarily affects children and is associated with systemic inflammation, particularly in the coronary arteries. Coxsackievirus A6 (CVA6) has emerged as a significant agent in atypical presentations of hand, foot, and mouth disease (HFMD), raising the possibility of its involvement in KD. **Case Presentation:** This report presents the case of an 18-month-old Thai boy admitted with symptoms of high fever, sore throat, and ulcerative lesions, initially diagnosed with herpangina. As his condition progressed, additional KD symptoms developed, including conjunctival injection, rash, and elevated inflammatory markers, fulfilling the diagnostic criteria for KD. Notably, throat swab analysis confirmed CVA6 as the causative agent. Phylogenetic analysis revealed that the CVA6 strain closely aligned with Chinese strains from 2023, showing a high nucleotide sequence homology of 98.4%. **Conclusions:** In conclusion, this case highlights a possible association between CVA6-associated herpangina and KD, suggesting that CVA6 infection may act as a trigger for KD in genetically susceptible children. These findings highlight the need for increased awareness among healthcare providers to promptly identify and manage Kawasaki Disease during peak enterovirus seasons, reducing its impact on children.

## 1. Introduction and Clinical Significance

Kawasaki disease (KD, mucocutaneous lymph node syndrome), a rare inflammatory disorder primarily affecting children under the age of 5, was first described by Dr. Tomisaku Kawasaki in 1967 [1]. The disease is characterized by the inflammation of medium-sized arteries, particularly coronary arteries. It can also involve the lymph nodes, skin, and mucous membranes [2]. Although the etiology of KD remains unknown, KD is thought to be triggered by an abnormal immune response to an infectious agent in genetically predisposed individuals [3]. Numerous studies have attempted to identify a specific pathogen responsible for the disease, but none have been conclusive [4,5,6,7]. The diagnosis of KD is clinical, based on a set of criteria defined by the Japanese Circulation Society Joint Working Group (JCS/JSCS) 2020 Guideline. These include fever, bilateral bulbar conjunctival injections, changes to the lips and oral cavity, rash, changes in the peripheral extremities (reddening of palms and soles, edema), and nonsuppurative cervical lymphadenopathy [8]. Early diagnosis and treatment with intravenous immunoglobulin (IVIG) are critical to prevent serious cardiovascular complications, such as coronary artery aneurysms [9].

Previous studies have reported a concerning rise in the global incidence of KD, observed in over 60 countries across Asia, the Middle East, the Americas, Africa, and Europe [10,11]. The highest incidence of KD is observed in Japan, followed by South Korea and Taiwan [12,13,14]. This trend underscores the need for increased awareness and further investigation into the underlying factors contributing to the increasing prevalence of this condition.

Coxsackievirus A6 (CVA6) has recently emerged as the leading genotype linked to global outbreaks of atypical hand, foot, and mouth disease (HFMD) and/or herpangina [15,16,17]. In Thailand, CVA6 has driven recurring large-scale HFMD outbreaks since 2012, solidifying its role as a major pathogen [18,19]. Our case presented with symptoms of both herpangina and KD, highlighting the potential comorbidity between CVA6-associated herpangina and Kawasaki disease, which this report seeks to investigate.

## 2. Case Presentation

**Figure 1:**
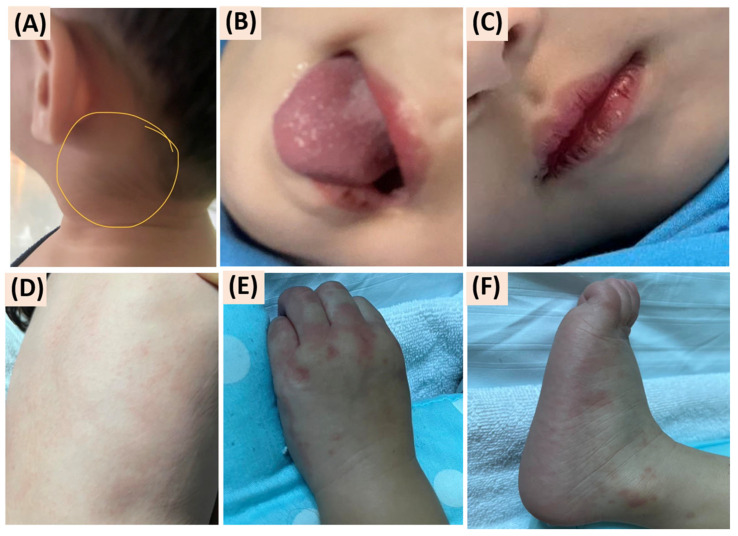
Clinical presentation of Kawasaki disease (KD): (**A**) cervical lymphadenopathy; (**B**) oral mucosal changes, including strawberry tongue; (**C**) fissured lips; (**D**) skin rash; and erythema with swelling of the (**E**) palms and (**F**) soles.

An 18-month-old Thai boy was admitted to the hospital with symptoms of high fever for one day before admission. He had a sore throat, ulcerative lesions on the soft palate, and swelling of enlarged cervical lymph nodes measuring 2.5 cm in diameter on both sites, with a temperature of 40.0 °C. The initial provisional diagnosis was herpangina. By the second day, the patient displayed dry lips, conjunctival injection, and redness and swelling of both palms and soles. A maculopapular rash developed on the trunk during the febrile period, accompanied by pharyngeal injection, ulcers on the soft palate, and a strawberry tongue (Figure 1). A cardiac examination revealed tachycardia without murmurs, lung auscultation was clear, and the abdomen showed no signs of hepatosplenomegaly.

The laboratory findings revealed a hemoglobin level of 10.1 g/dL, an elevated white blood cell count of 18,880/cumm3 (with 43% neutrophils, 48% lymphocytes, and 9% monocytes), a platelet count of 487 × 10^9^/L, and a C-reactive protein level of 22.17 mg/L (0.00–5.00 mg/L). The biochemistry test included BUN 3.7 mg/dL, creatinine 0.17 mg/dL, protein 7.4 gm%, albumin 4.1 gm%, globulin 3.3 gm%, direct bilirubin 0.2 gm%, total bilirubin 0.5 gm%, AST 35 unit/L, ALT 19 unit/L, and alkaline phosphatase 211 unit/L. Tests for coronavirus disease 2019 (COVID-19), influenza virus, adenovirus, and Respiratory Syncytial Virus (RSV), conducted using a nasopharyngeal swab, and hemo culture were all negative.

By the third day, echocardiography showed minimal pericardial effusion with normal cardiac structure and preserved ventricular function. The C-reactive protein level was 64 mg/L (0.00–5.00 mg/L) and the erythrocyte sedimentation rate (ESR) was 109 mm/h (0–10 mm/h). The patient was treated with intravenous immunoglobulins (IVIGs) on the fourth day of fever at a dosage of 2 g/kg administered with a good response within 24 h after treatment.

**Figure 2:**
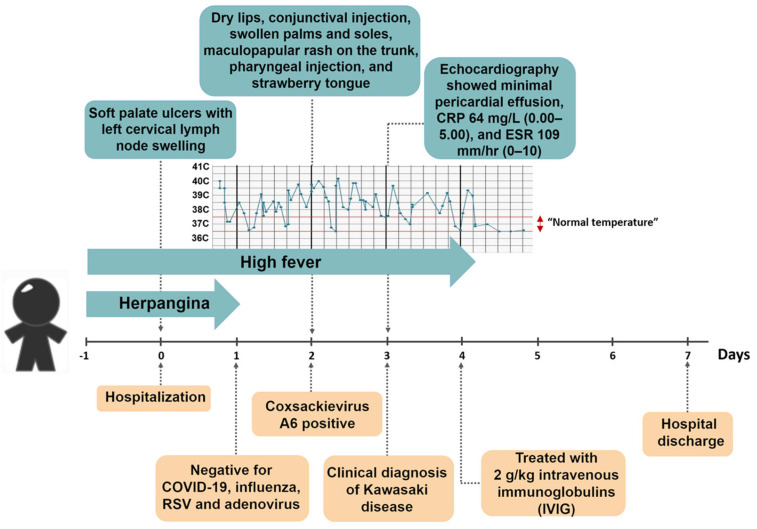
Timeline illustrating the progression of Kawasaki disease symptoms and corresponding treatments administered from onset until hospital discharge.

According to the JCS/JSCS 2020 Guideline [8], complete KD is diagnosed when at least four clinical features are present; other febrile illnesses are excluded. In this case, the patient met five of the criteria for complete KD (Figure 2), which included (1). bilateral bulbar conjunctival injection, (2). changes to the lips and oral cavity (reddening of lips and strawberry tongue), (3). rash, (4). edema and reddening of palms and soles, and (5). nonsuppurative cervical lymphadenopathy.

Following the initial diagnosis of herpangina, a throat swab was collected for further testing. The sample tested positive for enterovirus through pan-enterovirus detection, and CVA6 was identified using specific probe-based real-time RT-PCR [18]. A timeline depicting the progression of KD symptoms alongside the treatments administered from onset until hospital discharge is shown in Figure 2.

### Coxsackievirus A6 Detection

To characterize enteroviruses, our laboratory received a throat swab sample, from which we extracted viral RNA using the magLEAD 12gC instrument (Precision System Science, Chiba, Japan), following the manufacturer’s instructions. The viral RNA was tested for CVA6 through two separate real-time PCR reactions. First, each sample was subjected to real-time reverse transcription PCR (RT-PCR) for broad enterovirus screening, using primers and probes targeting the highly conserved 5’ untranslated region (UTR) of the enteroviral genome, as previously described [18]. Following a positive 5′ UTR result, a second real-time RT-PCR assay was performed with specific primers and probes targeting the VP1 region for precise CV-A6 typing [18]. Nearly complete genome amplification was performed using nested PCR with seven primer sets (Table 1), like a previously established protocol [19]. All amplicons were sequenced using the Sanger method and the resulting nucleotide sequences were deposited in the GenBank database under accession number PQ439203. Sequencing was performed on an Applied Biosystems 3730xl DNA Analyzer at Apical Scientific Sdn Bhd, Seri Kembangan, Selangor Darul Ehsan, Malaysia, using the BigDye Terminator v3.1 Cycle Sequencing Kit (Thermo Fisher Scientific, Waltham, MA, USA). We assembled and manually edited nucleotide sequences using Sequencher v.5.1 (Gene Codes Corporation, Ann Arbor, MI, USA). The sequences obtained were then aligned with CVA6 sequences from the GenBank database using CLUSTAL W on the European Bioinformatics Institute’s web server.

**Figure 3:**
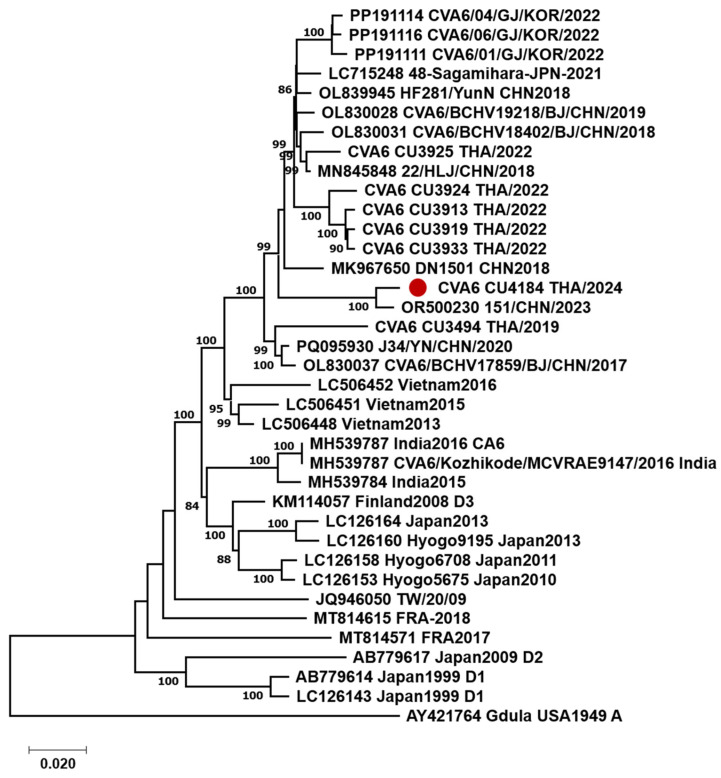
Unrooted phylogenetic tree of CVA6 variants based on nearly complete genome sequences. Bootstrap values (%) are shown for key nodes, derived from 1000 replicates. The highlighted CVA6 variant is indicated with a red circle. Scale bars denote substitutions per site.

A phylogenetic tree was generated using MEGA software (v10), employing the maximum likelihood method with the Kimura 2-parameter model. Bootstrapping was performed with 1000 replicates to ensure statistical reliability (Figure 3). Phylogenetic analysis of the 37 nearly complete genomes showed that the CVA6 strain from this study closely clustered with strains reported from China in 2023, sharing a high nucleotide sequence homology of 98.4%. This CVA6 strain showed nucleotide sequence homology with Thai strains from 2022, ranging from 93.7% to 99.5%, as well as with the Gdula prototype strain, at 80.7%.

## 3. Discussion

Despite being recognized for nearly 60 years, the precise cause of KD remains unclear. A widely accepted hypothesis suggests that a viral trigger induces an excessive inflammatory reaction in genetically predisposed children, though no particular pathogen has been identified [20]. In Thailand, enterovirus infections, including CVA6, generally follow a seasonal pattern, peaking between June and August during the rainy season [21]. Our case suggests that herpangina caused by CVA6 could potentially act as a trigger for the onset of KD, as observed in late August 2024, following the peak of infections during June and July. This aligns with a previous study showing that KD emerged two weeks after the peak of the COVID-19 epidemic, supporting the post-infection mechanism implicated in the development of KD [22]. The CVA6 strain identified in this study clustered with strains from China in 2023, suggesting it may have emerged from recent transmission events. This strain formed a distinct clade separate from Thai strains in 2022 and recombinant strains from 2019, indicating independent evolutionary pathways and highlighting the genetic diversity among CVA6 strains in Thailand. The association of this CVA6 strain with herpangina and hand, foot, and mouth disease (HFMD) may also contribute to the development of KD.

Children with siblings who have KD are at a significantly higher risk of developing the condition themselves, suggesting a possible genetic susceptibility linked to both sibling and parental history [23,24]. Previously, a case was reported of a 3-year-old boy who had both CVA6-associated HFMD and KD simultaneously. His older sister had also suffered from KD at the age of three, but she did not experience any concurrent illnesses [4]. The absence of concurrent illnesses, in this case, may be attributed to individual variability in immune responses and suggests that certain genetic factors may influence the manifestation of KD, allowing it to occur without the presence of additional infections or illnesses. A recent study in Taiwan observed a rising trend in both the annual incidence and seasonal patterns of KD, highlighting a notable correlation between KD cases in infants and the activity of prevalent respiratory and enteric viruses [25]. Documented associations between KD and viral infections, such as adenovirus, further supported this relationship [26].

The primary treatment for KD with established benefits in preventing coronary artery abnormalities is intravenous immunoglobulin (IVIG) therapy [27]. As evidenced in our case, IVIG treatment was most effective when administered on the fourth day of symptom onset, as indicated by a positive response.

Infections associated with KD, such as adenovirus, coronavirus, and influenza, typically manifest with respiratory symptoms and often lack the significant skin and mucosal involvement seen in CVA6 infections [22,26,28]. It is plausible that CVA6 infections, characterized by their distinct skin and mucous membrane manifestations, may more frequently overlap with the clinical features of KD compared to these other pathogens. In addition to viral infections, bacterial infections, including *Yersinia*, have been implicated in the pathogenesis of KD [29,30]. A previous study indicated that while *Yersinia* infection can present with symptoms similar to those of KD, routine screening for Yersinia is not warranted for all KD patients. Such screening should be reserved for individuals in high-risk areas or those who do not respond to standard KD treatment [31]. In this particular case, the absence of abdominal symptoms or signs in the patient led to the decision not to conduct *Yersinia* testing.

Numerous studies suggest that viral infections, particularly those caused by HFMD-associated viruses, may play a role in triggering KD in children. For instance, a 2012 Italian study reported two children with KD associated with CVB3 infection, both of whom showed coronary artery changes but experienced no long-term cardiologic effects [32]. A population-based cohort study in Taiwan also showed a significantly higher cumulative incidence of KD in individuals with enterovirus (EV) infections compared to those without [33]. Research from another group in Taiwan indicated that a reduction in KD admissions was closely associated with a decline in severe EV cases [34]. The rate of EV detection in the upper respiratory tract of KD patients was found to be four times higher than that in healthy children of the same age [35].

## 4. Conclusions

This case report highlights the relationship between CVA6 and KD, suggesting that CVA6-associated herpangina may trigger KD in genetically predisposed children. Our findings support the idea that viral infections can precipitate KD, underscoring the need for vigilant monitoring of enterovirus activity, especially during peak seasons. With the increasing global incidence of KD linked to various viral infections, including CVA6, healthcare providers must maintain heightened awareness for timely diagnosis and treatment. Early intravenous immunoglobulin (IVIG) intervention is crucial for preventing serious cardiovascular complications, highlighting the need for further research into the relationship between infectious agents and KD to enhance patient outcomes.

## Figures and Tables

**Table 1:** Primer set for full genome amplification of CVA6 using nested RT-PCR.

No.	Primer	Sequence (5′ to 3′)	Position
1	CVA6-F39/OS	ACT GGG CGC YAG CAC ACT GAT TC	39–61
	CVA6-R1673/OAS	AGT TAR TGT RAT TGG YAC YTC TGT	1650–1673
	CVA6-F55/IS	CTG ATT CTA YGG AAY CTT TGT GCG	55–78
	CVA6-R1567/IAS	GGA AGY GCR TTR ACA TAT GGC AT	1545–1567
2	CVA6-F1266/OS	TCG GGY TTC TGY ATG CAY GTT CA	1266–1288
	CVA6-R2806/OAS	GAY AGT TCT AGY TTG CGC CGC TG	2784–2806
	CVA6-F1290/IS	TGY AAY GCR AGC AAR TTC CAT CA	1290–1312
	CVA6-R2727/IAS	CCG AGT CCT TYA CCT CCA CAA C	2706–2727
3	CVA6-F2458/OS	CRA ATG CDG TGG AAA GYG CTG T	2458–2479
	CVA6-R3832/OAS	CCT TTG ATR TAA TCW GAY ACD CC	3810–3832
	CVA6-F2485/IS	GCR CTY GCT GAY ACC ACA ATA TC	2485–2506
	CVA6-R3816/IAS	GAC ACC CTG YTC CAT RGC TTC	3795–3816
4	CVA6-F3498/OS	GCT CAR GGA TGT GAY ACY ATT GC	3498–3520
	CVA6-R4564/OAS	CTA GAG TGR TAY TTR TCR GCT AT	4542–4564
	CVA6-F3603/IS	GTC TTY GTG GAA GCT AGT GAG TA	3603–3625
	CVA6-R4463/IAS	ACG GTG TTT GCT CTT GAA CTG CAT	4440–4463
5	CVA6-F4107/OS	AGY GCA TCN TGG CTH AAG AAG TT	4107–4129
	CVA6-R5482/OAS	TGA TCY GTY TGV ACY TGC CTR AT	5460–5482
	CVA6-F4214/IS	TRT ACC AGC AGC TAA AGA GAA GGT	4214–4237
	CVA6-R5330/IAS	GTA GAT RAC ATA CAC CAR TGA RAC	5307–5330
6	CVA6-F4994/OS	ATC CAA RGT BAG RTA YAG TGT GGA	4994–5017
	CVA6-R6422/OAS	GAG RTC AAR DCC ATA CTT RTC CAT	6399–6422
	CVA6-F5061/IS	GCY ATT GGN AAC ACA ATC GAA GC	5061–5083
	CVA6-R6364/IAS	GGG TCY AAR ATG TCY CTC TTC TT	6342–6364
7	CVA6-F6107-OS	CGC CTC GAG GTR GAT TTY GAR CA	6107–6129
	CVA6-R7428-OAS	CTG GTT ATA ACA AAT TTA CCC CCA CC	7403–7428
	CVA6-F6188/IS	GAR GCA GCH CTV CAY TAT GCA AAY CA	6188–6213
	CVA6-R7388/IAS	CCA GAT TYC TGG TGG GGT TGA G	7367–7388

## Data Availability

The GenBank accession number for the newly generated sequence obtained in this study is PQ439203.
